# Relationship between Provider Communication Behaviors and the Quality of Life for Patients with Advanced Cancer in Saudi Arabia

**DOI:** 10.3390/curroncol28040253

**Published:** 2021-07-30

**Authors:** Aisha Alhofaian, Amy Zhang, Faye A. Gary

**Affiliations:** 1 Medical Surgical Nursing Department, Faculty of Nursing, King Abdul Aziz University, P.O. Box. 80209, Jeddah 21589, Saudi Arabia; 2 Frances Payne Bolton School of Nursing, Case Western Reserve University, 10900 Euclid Ave., Cleveland, OH 44106, USA; Amy.Zhang@case.edu (A.Z.); faye.gary@case.edu (F.A.G.)

**Keywords:** provider communication behavior, quality of life, cancer, Saudi Arabia

## Abstract

**Context:** Patients with advanced cancer from Saudi Arabia are often not well informed about diagnoses, prognoses, and treatment options. Poor communication can lead to health-care decisions that insufficiently meet patients’ preferences, concerns, and needs and that subsequently affect patients’ quality of life. **Objectives:** The purpose of this study is to examine the relationship between provider communication behaviors and the quality of life of patients with advanced cancer. **Method:** A cross-sectional, correlation design was used in the present study, in which 159 patients with confirmed diagnoses of stage III or IV solid cancer were surveyed. **Results:** The mean summary score of the patients’ quality of life was 57.31. We found a significant relationship between provider communication behaviors and patient quality of life (β = 0.18, b = 0.35, SE = 0.15, *p* = 0.021). In addition, R2 shows that only 3.4% of variance in patient quality of life is predicated on provider communication behaviors. **Conclusions:** The relationship between provider communication behaviors and patient quality of life was low (*r* = 0.18). A possible reason for this is that provider communication behaviors are not the only factor that affects patient quality of life; other variables, such as the patient’s age, cancer type, and level of awareness, can also have an effect. Another possible explanation is that communication behaviors between patients and providers may vary depending on the level of cultural contact.

## 1. Introduction

Cancer is a devastating world-health problem. Saudi Arabia is a vast country in which the incidence of cancer has increased throughout the past several years. The Saudi Cancer Registry documented 27,885 cases in 2020 alone. In addition, the most common type of cancer in men is colorectal cancer, with a prevalence of 19.3% while in women it is breast cancer, with a prevalence of 29%. In Saudi Arabia, it is estimated that cancer incidence will rise between five- and tenfold by 2030. This will occur because of changing demographic characteristics. Specifically, middle-aged and elder populations are most affected by cancer [1].

Cancer and other life-threatening diseases need highly specialized caregivers who are experts in detecting and treating these maladies. A health-care provider is the first person who work directly and communicate with patients and their families about treatment plans and, at times, the expected health outcomes. Effective communication about the prognosis and treatment choices is an essential element to ensure that patients can make informed choices about treatment, can follow the provider’s advice about management, and can make appropriate adjustments to the fact that they have a life-threatening disease. In an oncology setting, cancer patients have multiple needs and concerns, including understanding information about their illness and the various treatment options, needing support when making decisions, and getting help with achieving behavioral change [2]. Moreover, patients who are more involved in communication and decision-making have better health outcomes [3], indicating a strong relationship between provider communication behaviors and the patient’s understanding of the treatment regimen [4].

Provider communication behaviors are defined as “a combination of verbal and action-related behaviors performed by physicians” [5]. These behaviors reveal doctors’ competence to effectively communicate with patients. There are two key elements of provider communication behaviors: verbal and nonverbal communication. Successful communication includes effectively providing information and asking questions; showing empathy, concern, or compassion; and creating a partnership in treatment [4]. The aim of such communication is to create a strong interpersonal relationship between providers and patients [6,7].

Patients from Saudi Arabia with advanced cancer may experience a number of critical challenges that are not evident in other countries, particularly when they involve communication and shared decision-making. In this culture, obtaining and discussing information about an individual’s illness is a family responsibility because it is the family members who use that information to make decisions about the patient’s best interests. Thus, family unity is important in communicating, making medical decisions, and providing care to patients in Saudi Arabia.

Because cancer therapies are complex, age and gender may play a role in understanding the need for these treatments and how they will improve quality of life [8]. Improved communication with family members, regardless of age or gender, is important so that patient-based preferences are recognized regarding the treatment plan [8]. In Saudi Arabian culture, female patients who are communicating with providers, especially male providers, tend to use a conservative approach, often acting passively and not seeking clarification or asking questions [9].

In addition, women’s male relatives (fathers, husbands, brothers, and sons) exercise decision-making powers over them as part of a cultural guardianship system. This systematic subordination of women is so pervasive within Saudi society that officials in many public and private institutions, including hospitals, require the permission of male relatives to offer services to women, even when there is no legal requirement to do so. For example, despite a 2012 regulation allowing females over 18 years of age to sign for admission or release from health care institutions, except in cases of abortion or sterilization, many officials still demand the signature of male relatives [10].

Prior studies have shown that patient quality of life and satisfaction with care are associated with positive outcomes in cancer care, and these outcomes can be influenced by effective provider communication behaviors [11]. Two reviews concluded that addressing patients’ emotional issues, partnership building, and provider informativeness were related to better physical, social, emotional, and functional well-being [12,13]. For example, when providers give patients enough information, the patients tend to experience less psychological distress and higher levels of symptom relief. Additionally, providers who encourage their patients to be active participants in their care and discuss goals for symptom control and intervention plans were linked to patients’ having better symptom control, lower levels of depression and anxiety, and decreased physical limitations. Furthermore, addressing patients’ emotional and social issues during the communication process was associated with improvements in physical and social status and overall health functioning. However, there is little known about the effect of patient-centered communication, specifically provider communication behaviors, on cancer-related quality-of-life outcomes. This study resolves a gap in the literature by examining the relationship between provider communication behaviors and the quality of life of patients with advanced cancer in Saudi Arabia.

## 2. Methods

### 2.1. Study Design

We used a cross-sectional, correlation design to explore the relationship between provider communication behaviors and the quality of life of patients with advanced cancer in Saudi Arabia.

### 2.2. Setting

Patients with stage III and stage IV gastrointestinal, genitourinary, breast, lung, or gynecological cancers were recruited from King Abdul-Aziz University (KAAU) Hospital in Jeddah.

### 2.3. Participants

The participants were 159 patients with confirmed diagnoses of stage III or IV solid cancer (gastrointestinal, genitourinary, breast, lung, and gynecological cancer) in Jeddah City, Saudi Arabia. Inclusion criteria were (1) ≥18 years old, (2) a diagnosis of stage III or IV solid cancer, and (3) being able to read and write in Arabic (the primary investigator would be able to read materials to low-literacy patients). Exclusion criteria included patients with cognitive impairment, such as psychosis or Alzheimer’s disease, because they might not be able to understand and complete the questionnaires. To determine if a person was cognitively impaired, the researcher administered the Arabic version of the Short Portable Mental Status Examination (SPMSQ). In the present study, a primary investigator was using cut-off score (5 errors) in answering the questions to determine the patient’s eligibility for to participate.

### 2.4. Variables

Patients completed Arabic versions of revised Physician-Patient Communication Behaviors (PPCB) scale and the European Organization for Research and Treatment of Cancer Quality of Life Questionnaire (EORTC QLQ-C30) (self-administered). In addition, we assessed demographic variables (age, gender, cancer type, educational level, awareness of terminal illness, and family income per month).

The revised Physician–Patient Communication Behaviors (PPCB) scale [14] contains a 13-item subscale that originated from the matched pair instrument (MPI), in which each item is evaluated on a 5-point scale, from 1 to 5 (1 = strongly disagree, 5 = strongly agree; [5]. The total scale score is 65; higher scores mean that the provider is more engaged in communication behaviors. The purpose of each item on the scale is to assess the provider’s effort, via communication, to encourage the patient to interact and to participate in the treatment plan. This scale defines the PPCB as “a combination of verbal and action-related behaviors performed by physicians” [5].

Internal consistency of the subscales was high for all items combined (mean alpha = 0.80). Factor loading supported construct validity of the 13-item PPBC scale [14]. Expert panel reviews supported face validity, and cognitive interviews supported content validity [14]. This scale measured patients’ perceptions regarding the providers’ communication behaviors. There was no Arabic version of this scale; therefore, the scale was translated into Arabic and pretested to evaluate the reliability and validity of the instrument.

The European Organization for Research and Treatment of Cancer Quality of Life Questionnaire (EORTC QLQ-C30) asks 30 questions to measure five functional subscales (physical, role, emotional, cognitive, and social functioning); three symptom scales (fatigue, nausea/vomiting, and pain); and five single items for remaining symptoms (dyspnea, insomnia, appetite loss, constipation, and diarrhea). It also measures the financial impact, global health status, and their quality of life.

The EORTC QLQ-C30 provides scores that range from 0 to 100; higher scores for the functional subscales and symptom scales reflect better function and worse symptoms, respectively. This instrument was reliable and valid in its Arabic version [15]. According to Cronbach’s alpha, its reliability has been estimated to be more than 0.70 for six out of nine subscales [15]. The lowest Cronbach’s alpha coefficients were for cognitive functioning (0.60), social functioning (0.65), and nausea and vomiting (0.43). It is low for nausea and vomiting because a low mean score for this subscale can be a result of the patients’ either not experiencing any nausea and vomiting or receiving good treatment [15].

### 2.5. Ethical Considerations

Institutional review-board approval was obtained according to the guidelines (Declaration of Helsinki). The primary investigator obtained written informed consent from all participants. In addition, each participant’s consent form was securely stored in a confidential file at the university, and the data collected from the questionnaires were kept separate and entered into a database that did not include personal identifying information. Each subject participating in the study was rewarded with a gift card for 40 S.R. in exchange for agreeing to participate in the study and complete the surveys.

### 2.6. Data Sources

The primary investigator obtained the informed-consent form from patients. Patients also received a copy of the informed consent and one letter that described the study, its purpose, and investigator’s information. Patients were asked to complete the PPCB scale and EORTC QLQ-C30 questionnaire along with their demographic data online on the Qualtirc Survey on an IPad at the hospital where they were being treated. The researcher has confirmed that the Qualtric Survey was available online from the internet in Saudi Arabia in places that insured comfort and privacy for the participants.

### 2.7. Study Size

The specified small-effect size was 0.10 for multiple regression statistical tests [16]. We used G*Power software to calculate the sample size. A statistical power analysis with an alpha of 0.05, power of 0.80, a moderately small-effect size of 0.10 and regression test with eight predictors suggested that at least 81 subjects were needed to constitute a statistically significant sample for multiple regressions. Thus, the current sample was adequate for detecting significant results for this analysis.

### 2.8. Statistical Methods

We used IBM SPSS Statistics 20 software for statistical analyses. We employed descriptive statistics to describe the categorical and continuous variables. We conducted simple linear regression analysis. The statistical level of significance was set at *p* < 0.05 for two sides.

## 3. Results

### 3.1. Pretest Results

The PPCB scale was translated from English to Arabic through the Certified Translation Center in Jeddah by a native Arab speaker who is fluent in Arabic and English. The translated instrument was then back-translated to English by two other translators at another certified translation center in Jeddah. Both English versions and the Arabic version were then compared for equivalency by two Saudi doctoral candidates. These procedures were repeated as necessary until the original PPCB scale and the back-translated versions were mutually agreed to be equivalent. Sixteen bilingual advanced-cancer patients were recruited to do the pretest; this represents 10% of the total sample in the full study. All 16 considered the contents of the Arabic version of the PPCB scale to be clear and understandable. Cronbach’s alpha coefficient for the Arabic version of the PPCB scale was 0.90. Arabic and English versions of the PPCB scale were significantly correlated (*r* = 0.99, *p* < 0.05).

### 3.2. Descriptive and Outcome Data

In the sample (Table 1), most participants (59.1%) were female. The participants’ ages ranged from 20 to 90 years (M = 54.55, SD = 15.02), and 74.2% (*n* = 118) of participants were married. In all, 37.1% of the patients had graduated from primary school, 14.5% had a secondary school education, 18.2% had a high school education, and 30% had a college education or greater. Most patients (*n* = 142, 89.3%) had never lived in another place for 3 or more months at one time, and 7.5% of the participants lived in Makkah. A majority of the participants (*n* = 128, 80.5%) were aware they had a terminal illness. Nearly 40% (*n* = 63, 39.6%) earned less than 3,000 SAR monthly from household income. The primary cancer types were varied, but the most common type (*n* = 32, 20.1%) was breast cancer, while the second most reported type was head and neck (*n* = 30, 18.9%). The mean summary score of participants’ quality of life was 57.31. The mean scores of eight symptoms’ subscales for their quality of life were 71.42, 44.55, 67.92, 18.66, 55.14, 55.14, 18.66, and 29.98 for FA, NV, PA, DY, SL, AP, CO, and DI, respectively (Table 2).

### 3.3. Main Results

We calculated simple linear regression to predict patient quality of life using provider communication behavior as a predictor. Significant regression equation was found (F(1158) = 5.46, *p* = 0.021), with R2 of 0.034. Participants’ predicted quality of life was equal to 38.798 + 0.354 (provider communication behaviors. In addition, R2 showed that only 3.4% of variance in patient quality of life was predicated by provider communication behaviors. There might be many factors that can explain this variation, but our model, which includes only provider communication behaviors, can explain approximately 3.4% of it. The other 96.6% of the total variation in patient quality of life remains unexplained.

Although only a small amount of variance in patient quality of life was explained by provider communication behaviors, the regression results showed that provider communication behavior significantly predicted patient quality of life β = 0.18, b = 0.35, SE = 0.15, *p* = 0.021. However, the direction of the relationship was positive: the more communication behaviors the provider engages in the better quality of life the patients will have.

## 4. Discussion

### 4.1. Statement of Principal Findings

Our findings indicated that there were significant relationships between provider communication behaviors and patient quality of life for advanced cancer patients in Saudi Arabia, which is consistent with findings of previous studies [12,17].We found that the relationship between provider communication behaviors and patient quality of life was low (*r* = 0.18). In terms of shared variance (R2 = 0.034), 3.4% of the total variation in patient quality of life can be explained by the linear relationship between provider communication behaviors and patient quality of life. The other 96.6% of the total variation in patient quality of life remains unexplained.

### 4.2. Strength and Limitations

This correlation study has some limitations. This type of design does not allow the researcher to manipulate and control the major independent variables. In addition, the cross-sectional design represents a finding that occurred at only one point in time for the cancer patients, so causality cannot be anticipated. In addition, the sample in this study represented participants only from King Abdul Aziz University Hospital in Jeddah, which may decrease the generalizability of its findings to all advanced cancer patients within and outside of Jeddah. Additionally, all of the study’s measurements were based on a self-report scale; for this reason, the possibility of reporting biases or recall biases cannot be eliminated. Moreover, this study was done on patients who were restricted to a public health-care system in which medical treatment choices were limited. Most were from poor to middle class families who did not complain about their quality of life. This hospital environment may have significantly affected our findings.

In addition, the regression model in the current study did not include medical and treatment variables such as cancer stage, symptom severity and comorbidity that can affect patient’s quality of life, nor providers’ sociodemographic characteristics such as gender or length of the patient–provider relationship that can affect provider communication behaviors. Whether all these factors are connected in some way remains to be explored. If this is the case, these relationships should be studied empirically. However, the current study is considered a preliminary study that was done for first time in Saudi society.

### 4.3. Interpretation

The relationship between provider communication behaviors and patient quality of life was low. A possible reason for this is that provider communication behaviors are not the only factors that affect patient quality of life; other variables, such as patient’s age, cancer type, and level of awareness, or provider’s characteristics (e.g., gender) can also have an effect. Another possible explanation is that communication behaviors between patients and providers may vary depending on the level of cultural contact. Most of the study’s participants were females who adhered to Islamic rules and followed restrictive cultural norms. A female patient cannot talk or meet with male doctors alone. If she does not have a relative who can stay with her, she needs to remain in the presence of either a female nurse or doctor. Some of them were able to establish a stronger reciprocal communication with their health-care providers than with their own family members. Others had family members take care of the communication with providers, something that made mutual understanding more complicated. Moreover, health-care providers may ask fewer biomedical and psychosocial questions, particularly when a family member is present, resulting in concerns about the length of the visit. Health-care providers may focus on building rapport with a patient and the family during their communication in an accompanied visit, which could lead to insufficient evaluation of patient concerns about biomedical issues [18]. Thus, a provider’s communication behaviors were irrelevant to the patient’s needs and concerns

Another possible explanation is that the patient-provider gender concordance may influence patients’ perception of providers’ communication behavior. A meta-analysis done by Roter et al. in 2002 concluded that female health- care providers tend to engage more than males in partnership-building communication behaviors, to seek additional information from the patient about psychosocial issues, to spend more time with patients, and to show more interest in discussing emotion. Moreover, female patients have reported greater satisfaction when female providers deliver their care compared to those who received care from male providers [19,20]. In this study, most of the participants were female who might have been seen or examined by male health care providers. The patient-provider gender concordance or discordance may explain why the relationship between provider communication behaviors and patient quality of life was relatively low in this study.

### 4.4. Implication for Policy, Practice, and Research

This study has several implications for how oncology nurses may work with patients, patients’ families, and other providers during assessment, intervention, and follow-up. Nurses should maintain ongoing communication with patients in a self-management and pain-control intervention because these activities influence the patients’ quality of life. In addition, there are significant differences between our study and other studies that can be explained by the important differences among patients’ characteristics, treatment options, palliative treatment, and culture norms in Saudi Arabia compared to those in studies conducted in Western countries. Due to the complexity of treatment choices, health- care providers in Saudi Arabia may not have multiple treatment options for advanced cancer patients; rather, they may have general treatment plans that might apply for most patients. Furthermore, there were no individualized treatments for patients that can meet each patient’s particular needs and concerns. Therefore, the quality of life of cancer patients in Saudi Arabia may be affected by many factors that have not yet been studied.

### 4.5. Conclusions

The purpose of our study was to examine the relationships between provider communication behaviors and patient quality of life in Saudi Arabian late-stage cancer patients. The study was a correlation analysis conducted in King Abdul-Aziz University Hospital in Jeddah. Our findings illustrate relevant features in modern Saudi Arabian society, such as the significant relationships among the study’s variables, future research is needed to study family or caregiver–provider communication so that long-term health outcomes can be maximized.

## Figures and Tables

**Table 1:** Demographic Characteristics.

Variables	*N*	%
Sex		
Male	65	40.9
Female	94	59.1
Marital status		
Single	16	10.1
Married	118	74.2
Separated	8	5
Widowed	13	8.2
Divorced	4	2.5
Education Level		
Primary school	59	37.1
Secondary school	23	14.5
High school	29	18.2
College and above	48	30.2
Total Income		
<3000 Saudi Arabian Riyals (SAR)	63	39.6
3000 to 5000 (SAR)	42	26.4
>5000 (SAR)	54	34
Awareness of Terminal Illness		
Yes	128	80.5
No	31	19.5

**Table 2:** Descriptive Statistics of Major Study Variables.

				Range			
Variables	N	M	SD	Possible	Actual	Skewness	Kurtosis
Age (Years)	159	54.55	15.02	20–90	20–90	−0.005	−0.298
(PPCB) scale overall score	159	52.24	10.92	13–65	13–65	−0.954	0.164
(EORTC QLQ-C30) Summary score	159	57.31	21.11	0–100	0–100	−0.116	−0.839
Global health status (QL2)	159	56.39	22.53	0–100	0–100	−0.463	0.009
Physical functioning (PF2)	159	46.79	34.07	0–100	0–100	−0.131	−1.39
Role functioning(RF2)	159	45.39	38.10	0–100	0–100	0.269	−1.39
Emotional functioning(EF)	159	62.21	31.74	0–100	0–100	−0.540	−0.845
Cognitive functioning (CF)	159	80.61	30.52	0–100	0–100	−1.46	0.979
Social functioning (SF)	159	71.49	34.60	0–100	0–100	−0.987	−0.372
Fatigue (FA)	159	71.42	30.50	0–100	0–100	−0.975	−0.253
Nausea and vomiting (NV)	159	44.55	39.59	0–100	0–100	0.103	−1.572
Pain (PA)	159	67.92	34.45	0–100	0–100	−0.847	−0.600
Dyspnea (DY)	159	18.66	35.10	0–100	0–100	1.62	0.983
Insomnia (SL)	159	55.14	41.76	0–100	0–100	−0.277	−1.57
Appetite loss (AP)	159	55.14	41.08	0–100	0–100	−0.279	−1.53
Constipation (CO)	159	18.66	34.70	0–100	0–100	1.58	0.873
Diarrhea (DI)	159	29.98	39.72	0–100	0–100	0.79	−1.08
Financial difficulties (FI)	158	17.30	33.59	0–100	0–100	1.65	1.11

Note. PPCB = Physician-Patient Communication Behaviors Scale; EORTC QLQ-C30 = European Organization for Research and Treatment of Cancer Quality of Life Questionnaire.

## Data Availability

Data used and analyzed in this study will be promptly available for the publisher upon request.
